# Optimizing online groundwater monitoring in industrial parks using long-term high-frequency data

**DOI:** 10.1016/j.isci.2026.117042

**Published:** 2026-08-06

**Authors:** Shuping Yi, Pizhu Huang, Yi Deng, Yi Liu, Hefei Huang, Zhiren Tian

**Affiliations:** 1 School of Environmental Science and Engineering, Southern University of Science and Technology, Shenzhen 518000, China; 2 MME Key Laboratory of Integrated Surface Water-Groundwater Pollution Control, Shenzhen 518000, China; 3 Department of Ecology and Environment of Jiangxi Province, Nanchang 330000, China; 4 China National Environmental Monitoring Centre, Beijing 100012, China

**Keywords:** groundwater environment, online monitoring, indicator optimization, monitoring frequency, pre-warning threshold

## Abstract

Chemical industrial parks require groundwater monitoring strategies that can detect abnormal changes while limiting redundant measurements. We analyzed nearly three years of hourly data from eight online monitoring wells in a chemical industrial park. Indicator screening combined the normalized interquartile range with Spearman correlation analysis; monitoring frequency was optimized using downsampling and reconstruction-error metrics; and predictive validation employed ridge and random forest regression. Online monitoring captured sudden and persistent anomalies lasting 100–3,360 h. Ammonia nitrogen (NH_3_-N), chemical oxygen demand (COD), turbidity, pH, and groundwater level were the most informative indicators. Optimized intervals ranged from 29 to 720 h and varied by well and parameter. Seasonal sensitivity analysis supported the robustness of moderately optimized intervals, and site-specific dynamic thresholds reduced excessive alarms under disturbed baseline conditions. The framework supports practical design of adaptive groundwater online monitoring and early warning in industrial parks.

## Highlights

• Nearly three years of hourly data revealed abrupt and persistent groundwater anomalies
• Variability and redundancy analyses identified informative online indicators
• Optimal monitoring intervals varied markedly among wells and parameters
• Seasonal validation and site-specific thresholds supported practical early warning

## Graphical abstract

**Figure:**
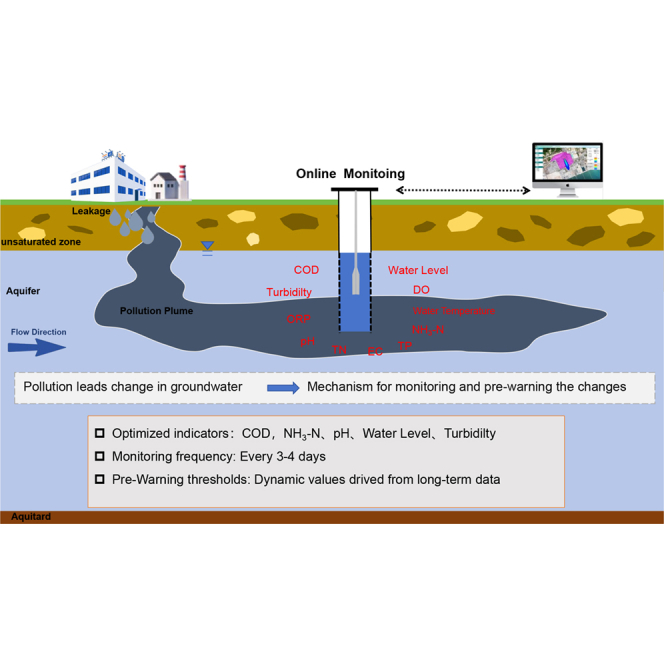

## Teaser

Environmental science; Environmental monitoring; Pollution

## Introduction

Groundwater resources are essential for global water security, supporting ecosystems, agriculture, and human activities worldwide.^1^ However, increasing demands on groundwater, particularly in regions with intensive industrial activities like chemical industrial parks, have rendered these critical resources increasingly susceptible to pollution and overexploitation.^2^ The groundwater environment in China has been significantly threatened by the widespread presence of industrial parks, which has received considerable nationwide attention.^3^^,^^4^^,^^5^^,^^6^

Due to slow recharge rates and the challenges of remediation once contamination occurs, groundwater systems are particularly vulnerable to pollution.^7^ *In situ* monitoring has emerged as an indispensable technique for assessing groundwater dynamics and quality. Continuous monitoring is crucial for understanding temporal changes within aquifer systems.^8^ Compared to traditional sampling, *in situ* monitoring provides continuous data collection, reduces labor costs, and enables the detection of rapid shifts in water quality parameters.^9^^,^^10^ High-frequency monitoring, in particular, can reveal short-term trends and fluctuations that conventional, low-frequency sampling might miss.^11^

Previous studies on groundwater monitoring optimization have mainly focused on the spatial design of monitoring networks, such as determining the optimal number and placement of monitoring wells.^12^^,^^13^^,^^14^ For example, a gamma test theory-based approach has been proposed for the optimal design of groundwater monitoring networks, with an emphasis on well selection.^15^ Similarly, gamma test theory has been integrated with a monitoring priority map to optimize well selection in groundwater monitoring networks.^16^ However, these studies primarily addressed the spatial configuration of monitoring systems rather than the optimization of temporal sampling frequency based on long-term, high-resolution observations.^17^^,^^18^ In contrast, the present study focuses on how hourly online groundwater monitoring data can be used to optimize monitoring frequency, evaluate indicator informativeness, and support dynamic early warning in an industrial park setting.^19^

Effective groundwater monitoring relies on selecting and analyzing appropriate indicators. Statistical methods help identify key indicators for urban groundwater contamination.^20^ Optimizing the frequency of monitoring is essential for balancing data quality with resource efficiency, and geostatistical analysis plays a pivotal role in refining temporal monitoring intervals.^21^ Establishing meaningful thresholds for groundwater indicators is essential for effective management and early warning systems.^22^^,^^23^ Systems that incorporate multiple indicators, such as groundwater quality, trends, and pollution risks, have proven effective for regional groundwater pollution early warning.^24^

Advancements in high-frequency and long-term *in situ* monitoring have significantly improved our understanding of groundwater systems.^6^^,^^25^^,^^26^^,^^27^ Integrating advanced sensors, optimized monitoring strategies, and sophisticated data analysis techniques has greatly enhanced groundwater resource management and protection.^28^^,^^29^^,^^30^^,^^31^ Future research should focus on refining monitoring methodologies, enhancing data interpretation, and developing adaptive management strategies based on real-time monitoring data.^32^ Integrating real-time monitoring data into environmental policy frameworks with adaptive management strategies is crucial.^8^

Although previous studies have demonstrated the value of high-frequency groundwater monitoring, three gaps remain. First, many studies focus primarily on describing temporal variation or contamination events, while fewer provide a systematic framework for jointly optimizing indicator selection, monitoring frequency, and warning thresholds for practical online monitoring systems. Second, the rationale for selecting an appropriate temporal sampling interval is often insufficiently quantified, particularly under long-term real-world monitoring conditions in industrial parks. Third, validation of frequency optimization results is still limited, and it remains unclear whether reduced sampling frequencies can preserve the temporal information needed for downstream prediction and early warning.

To address these gaps, this study uses nearly three years of hourly groundwater monitoring data collected from an industrial park in China to evaluate the practical performance of an online monitoring system. Specifically, this study aims to:

(1) assess the effectiveness of online monitoring for identifying groundwater anomalies associated with accidental or anthropogenic disturbances;
(2) optimize monitoring indicators by combining normalized interquartile range (NIQR) analysis and Spearman correlation analysis;
(3) determine an appropriate monitoring frequency using time-domain downsampling and error-based comparison;
(4) establish dynamic early warning thresholds based on long-term monitoring data. In addition, predictive validation using ridge regression and random forest regression was introduced to test the robustness of the optimized monitoring frequency.

The results provide practical guidance for the design and management of groundwater online monitoring systems in industrial parks. Compared with previous studies that mainly focused on descriptive monitoring or single-task optimization, this study provides an integrated framework that links indicator screening, monitoring-frequency optimization, predictive validation, and dynamic threshold setting within a real industrial-park groundwater monitoring system. It should be noted that the objective of this study was not to develop a fully automated machine-learning early-warning model. Instead, machine-learning models were used as independent predictive-validation tools to test whether the optimized sampling frequencies retained information useful for subsequent prediction. The development of machine-learning-based abnormal-event classification and fully adaptive threshold updating requires independently labeled event records and prospective operational validation, and is therefore discussed as a future extension of the proposed framework.

### Study design and monitoring dataset

#### Study area

The study area is located in a chemical industrial park with 37 evenly distributed enterprises, among which 17 are identified as the key contaminant discharge units, primarily involved in chemical raw material production, dye manufacturing, and textile processing. The primary contaminant source in the park is the wastewater from these key units, where inappropriate development practices and high pollutant emissions pose significant pollution risks to the underlying groundwater systems. Traditional management practices rely on manual sampling twice a year, which fails to capture the full extent of pollution. Groundwater pollution has occurred frequently in the area and led to adverse social impacts in the past few years.

Groundwater in the park area can be divided into overlying shallow pore water and underlying fissure water. Based on available data and on-site hydrogeological surveys, the shallow groundwater is primarily recharged vertically by atmospheric precipitation seepage and is most vulnerable to the contaminants produced by the densely distributed pharmaceutical and chemical enterprises on the surface. Groundwater levels in the park fluctuate seasonally with variations of approximately one meter. Groundwater flows mainly from the southwest to the northeast and discharges via evaporation and seepage into the river.

To capture real-time groundwater changes and improve the efficiency of groundwater contamination risk identification, early warning, and management, higher-frequency online monitoring is needed instead of traditional manual sampling conducted twice a year. Online monitoring reduces data gaps caused by the long intervals between traditional sampling, strengthening the scientific foundation for decision-making.^19^^,^^33^

Variations in the park’s groundwater environment stem from multiple pollution events confirmed by accidents and on-site sampling. These incidents have directly affected groundwater hydrodynamics and hydrochemical indicators within the chemical industrial park. Characteristic contaminants—such as ammonia nitrogen (NH_3_-N) and chemical oxygen demand (COD)—may rise immediately following a leak or indirectly through dilution and complex aquifer-scale hydrogeochemical processes. In all cases, it is evident that leakage from anthropogenic activities or accidental releases induces measurable responses in key groundwater indicators, which is the central focus of our monitoring and analysis.

**Figure 1:**
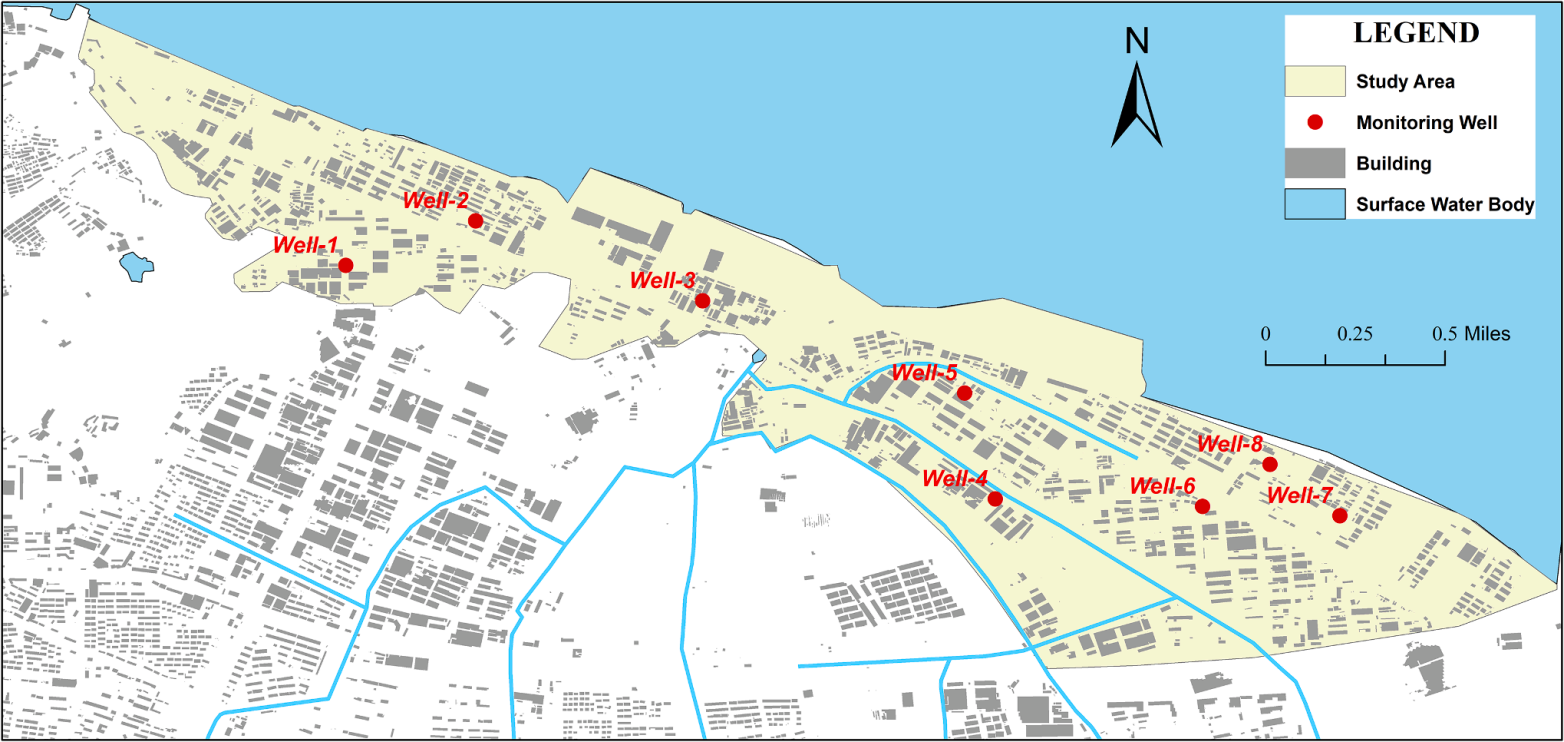
Location of the study area and the distribution of the eight online monitoring wells at the industrial park

Eight online monitoring wells were established for the shallow groundwater within the industrial park, based on groundwater recharge, runoff, and discharge characteristics, together with the distribution of contaminant sources and financial constraints. The eight online monitoring wells were strategically located downstream of potential pollution sources, as shown in Figure 1. The monitoring system was constructed over one year and subsequently generated nearly three years of continuous monitoring data.

These wells facilitate the online monitoring of the shallow groundwater environment for the industrial park. Eleven hydrodynamic and hydrochemical indicators, including water level, water temperature, pH, electrical conductivity (EC), turbidity, oxidation-reduction potential (ORP), dissolved oxygen (DO), COD, NH_3_-N, total nitrogen (TN), and total phosphorus (TP), were monitored hourly. Under standard operational conditions, the monitoring equipment is calibrated to record one data point per hour for each indicator. The selected indicators represent a combination of hydrodynamic and hydrochemical parameters that are widely used in groundwater monitoring and are technically feasible for stable online measurement in industrial-park settings. Water level and temperature were included to characterize hydrodynamic conditions, whereas pH, EC, turbidity, ORP, DO, COD, NH_3_-N, TN, and TP were selected to reflect physicochemical changes and organic- and nutrient-related pollution risks. The indicator set was designed to balance environmental relevance, sensor reliability, and operational cost. These indicators were selected not only because they are commonly used in groundwater quality assessment, but also because they are measurable with stable online sensors and directly relevant to the dominant pollution risks in chemical industrial parks.

Nevertheless, this online indicator set should not be interpreted as a complete list of all characteristic pollutants in the chemical industrial park. Trace organic pollutants and heavy metals are important site-specific contaminants, but they are generally monitored through periodic laboratory analysis rather than stable hourly online measurement. Therefore, the indicators analyzed in this study were used to characterize hydrodynamic disturbance, general hydrochemical variation, organic/nutrient-related pollution responses, and early abnormal changes. Comprehensive risk assessment in chemical industrial parks should further combine high-frequency online monitoring with targeted laboratory analysis of heavy metals, volatile/semi-volatile organic compounds, and other site-specific pollutants determined from production processes, raw materials, waste streams, and historical contamination records.

#### Data collection

Eight VLWS-505 automatic water-quality monitoring stations were deployed at the monitoring wells in the study area. Each station was equipped with a TNP-4200 online TP/TN analyzer and a TOC-4200 online total organic carbon analyzer. Sensors were installed within the phreatic zone of the unconsolidated porous aquifer through the screened sections of the wells. Details of instrument specifications, calibration procedures, measurement uncertainty, data-quality screening, and laboratory verification are provided in STAR Methods and Table S1.

The monitoring period extended from January 22, 2021, to November 13, 2023, with automatic hourly sampling used to capture real-time groundwater-quality dynamics in the industrial park. Despite minor interruptions related to power outages and instrument maintenance, the online monitoring system yielded 1,794,813 hourly observations. Details of the monitored indicators, monitored wells, observation numbers, and units are provided in Table 1.

**Table 1:** Summary of the hourly monitoring dataset for the groundwater indicators collected from the eight monitoring wells

Indicator	Number of monitored wells	Number of observations	Unit
COD	8	260864	mg/L
ORP	8	260864	mV
TN	2	65216	mg/L
pH	8	260864	dimensionless
TP	8	260864	mg/L
NH_3_-N	8	260864	mg/L
Water level	8	260864	m
Water temperature	8	260864	°C
Turbidity	8	260864	NTU
DO	8	260864	mg/L
EC	8	260864	μS/cm

To protect the confidentiality of raw concentration values, monitored time series are shown as robust-scaled values in the figures. Unless otherwise stated, statistical analyses, indicator screening, monitoring-frequency optimization, predictive validation, and threshold calculation were performed using the quality-controlled monitoring data rather than the visualization-scaled values. This transformation preserves relative temporal trends and fluctuations without disclosing site-specific raw concentration values. The robust-scaling procedure is described in STAR Methods.

**Figure 2:**
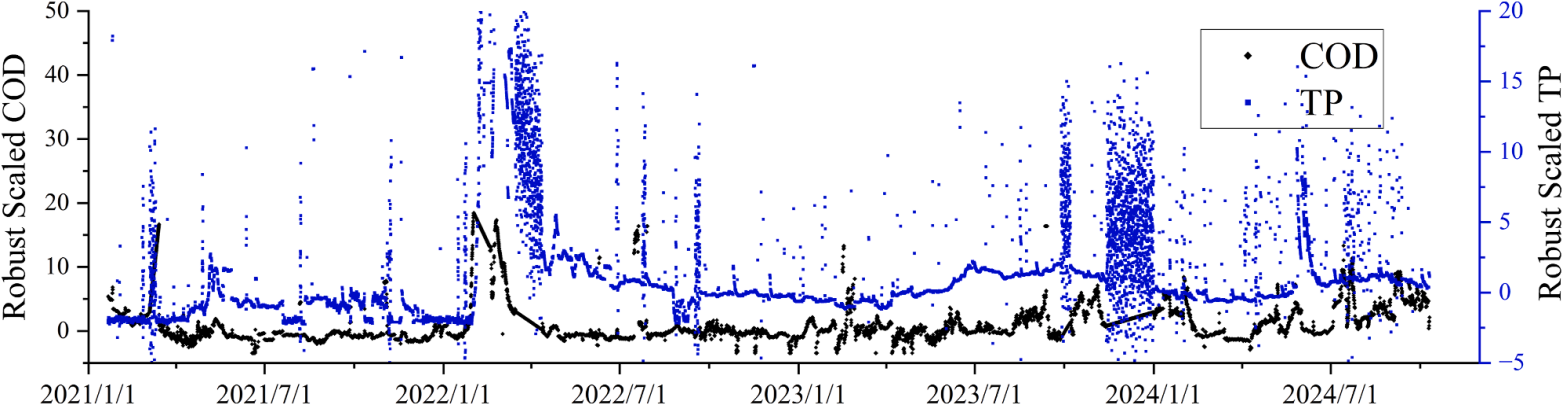
The data series of COD and TP obtained in Well-5 The blue trace delineates TP, and the black trace corresponds to COD. Values on the vertical axis are shown as robust-scaled values for visualization.

Figure 2 compares the COD and TP time series observed at Well-5. Examination of the data reveals that the initial phase demonstrates discordant oscillations, while the subsequent phase shows a more consistent trend. Notably, these indicators show varying degrees of fluctuation similarity, suggesting distinct response mechanisms to environmental changes.

## Results

### Time-series analyses

**Figure 3:**
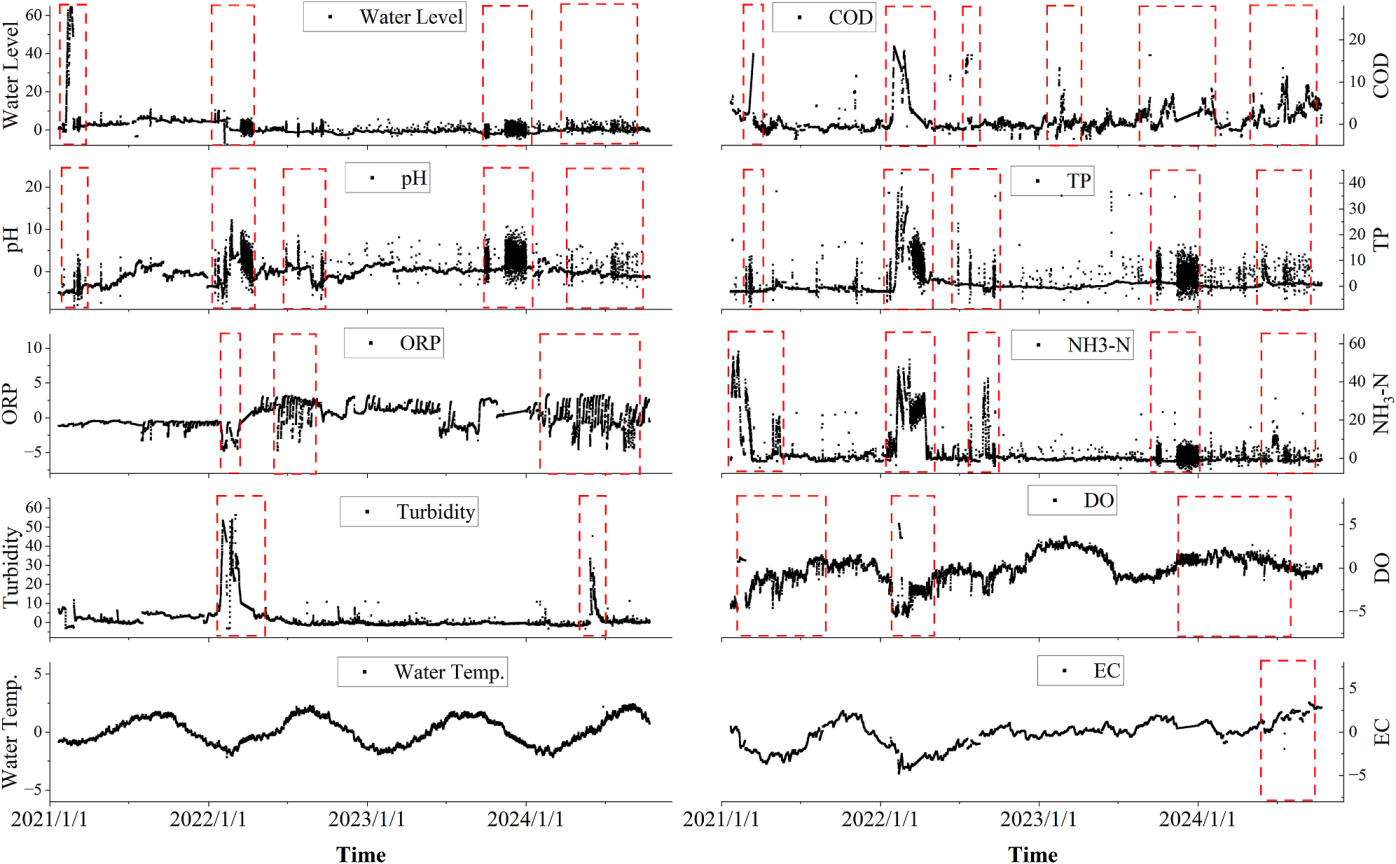
Online hourly monitoring results for Well-5 The scatterplots show the relationships among pH, TP, NH_3_-N, and groundwater level. The red background matrix highlights the duration of evident abnormal changes in groundwater indicators. Values on the vertical axis are shown as robust scaled values.

Figure 3 presents the online hourly time series of the 10 indicators monitored at Well-5. Water temperature exhibited the clearest seasonal pattern, and groundwater level also showed wet-dry period fluctuations associated with recharge conditions. By contrast, the seasonality of most hydrochemical indicators was less pronounced and was frequently superimposed by irregular short-term or persistent fluctuations related to hydrological disturbance and potential anthropogenic inputs. Therefore, both seasonal background variation and event-related abnormal changes were considered in the subsequent interpretation and validation. In addition to these seasonal fluctuations, several abrupt changes persisted for approximately 100 to 3,360 h, which could be identified through the combined interpretation of the time-series plots and the abnormal-duration statistics. These results suggest that online monitoring data can effectively distinguish natural variations from potential anthropogenic disturbances, thereby enabling the identification of abnormal periods. Compared with conventional manual sampling, online monitoring provides more continuous and higher-resolution observations, allowing more accurate reconstruction of actual groundwater environmental conditions.

Figure 3 also shows that the long-term monitoring record over approximately 34 months enabled the identification of real-time anomalies in the groundwater environment. The coordinated responses of hydrodynamic and hydrochemical indicators strengthened the interpretation of groundwater environmental changes. In particular, fluctuations in groundwater level were often accompanied by variations in hydrochemical indicators such as NH_3_-N, COD, and TP, suggesting possible hydrological and geochemical linkages. However, changes in hydrochemical indicators did not always induce obvious shifts in hydrodynamic parameters. Such coupled variations may reflect dilution-concentration effects, changes in hydraulic gradients, pollutant leaching following accidental releases, and subsequent hydrogeochemical reactions within the shallow aquifer. Specifically, groundwater-level fluctuations can alter flow paths and residence time, thereby affecting the transport and apparent concentrations of hydrochemical indicators. Therefore, integrated interpretation of hydrodynamic and hydrochemical responses is essential for distinguishing natural seasonal variability from potential anthropogenic disturbances.

The observed coupled responses can be further explained by the hydrogeological setting of the industrial park. The shallow groundwater is mainly recharged by atmospheric precipitation and is vulnerable to surface-derived contamination from densely distributed chemical and pharmaceutical enterprises. Therefore, groundwater-level rises may influence water quality through two opposite processes. On the one hand, recharge can dilute dissolved constituents and reduce the apparent concentrations of relatively conservative indicators such as conductivity. On the other hand, rapid infiltration can mobilize pollutants accumulated in shallow soils or the vadose zone and transport them into groundwater, leading to short-term increases in NH_3_-N, COD, TP, and turbidity.

In addition, groundwater-level fluctuations can modify local hydraulic gradients, flow paths, and residence time, thereby affecting the migration, dilution, and attenuation of pollutants in the shallow aquifer. This mechanism explains why some abnormal hydrochemical responses were synchronized with groundwater-level changes, whereas others occurred without obvious hydrodynamic shifts. Indicators such as NH_3_-N, COD, TP, and turbidity are more directly related to pollutant input, particle mobilization, and organic/nutrient enrichment, whereas pH, DO, and ORP are more strongly influenced by acid-base buffering and redox reactions. In particular, the negative association between NH_3_-N and ORP suggests that nitrogen-related pollution may be accompanied by more reducing conditions caused by organic matter degradation and oxygen consumption. These process-based interpretations indicate that coupled hydrodynamic and hydrochemical analysis can improve the discrimination between natural seasonal variability and anthropogenic disturbance.

After excluding changes mainly attributable to natural factors, the abnormal variations associated with anthropogenic influences were found to persist for approximately 100 to 3,360 h. This result indicates that the groundwater online monitoring interval should be shorter than 100 h to avoid missing transient but environmentally significant changes caused by potential pollution incidents. Overall, online monitoring provided greater temporal continuity and resolution than conventional manual sampling, enabling the detection of both natural and anthropogenic changes in groundwater indicators. Integrated analysis of multiple indicators further improved the identification of abnormal periods and could improve the information basis for early warning. These findings highlight the importance of online monitoring and provide practical guidance for selecting effective warning indicators and appropriate monitoring frequencies for groundwater environmental management in chemical industrial parks.

### Indicator optimization

**Figure 4:**
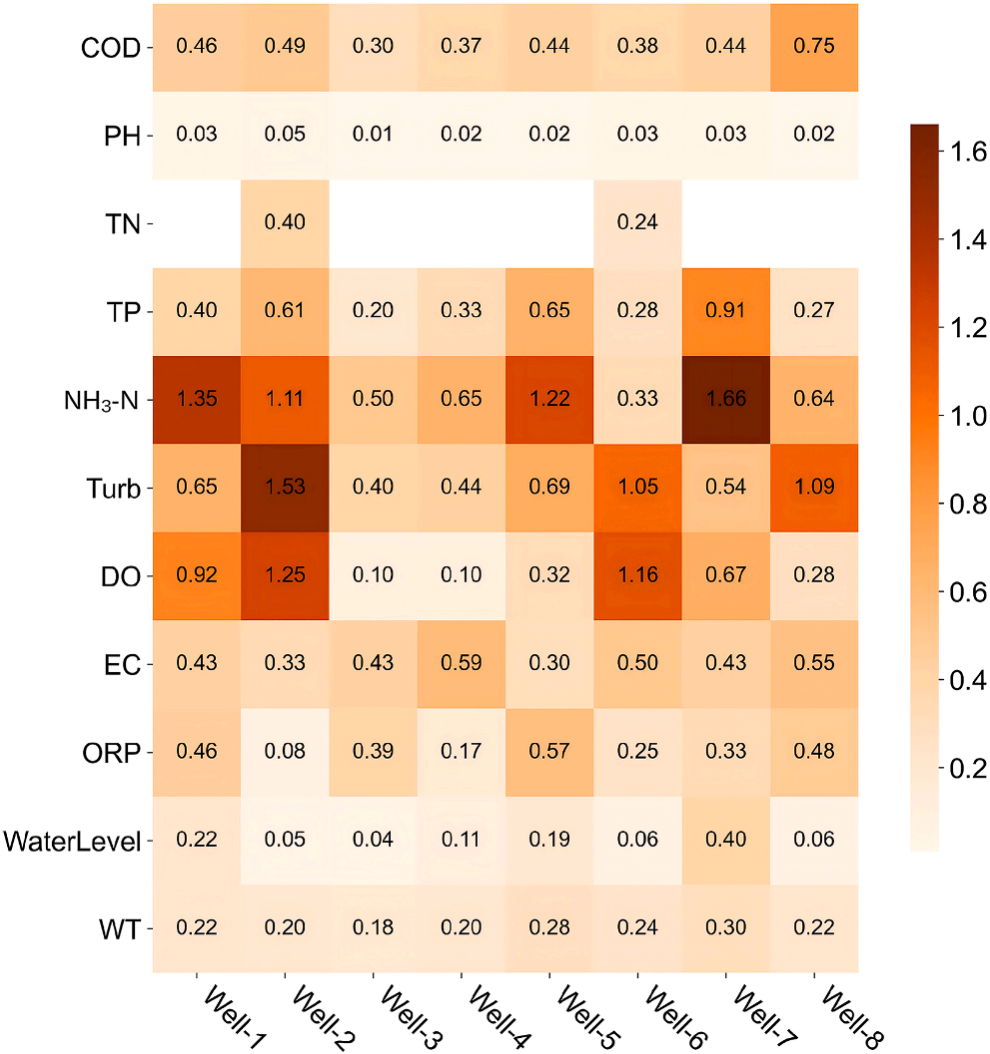
The normalized interquartile range (NIQR) of various indicators at 8 monitoring wells

Figure 4 shows the NIQR for the data of 11 monitored indicators across monitoring wells (Well-1 to Well-8). NIQR is calculated after data cleaning and classification to analyze the degree of variation in the indicators at different wells. Higher NIQR values indicate greater variability, while lower values suggest stability.

NH_3_-N and turbidity exhibited substantial variability across wells, whereas pH, water temperature, and groundwater level were comparatively stable. COD and TP also showed relatively high variability. The remaining indicators exhibited moderate or low variability and were therefore less informative in the variability-based screening.

The NIQR values calculated for different wells provide useful information for the spatial management of online monitoring. It can be seen that the calculated NIQR values were higher at Well-1, Well-2, Well-5, Well-6, and Well-7, indicating that significant groundwater changes occurred in the areas surrounding these wells. The NIQR values calculated for Well-3, Well-4, and Well-8 show relatively low variability, indicating that the groundwater in these areas is relatively stable and requires less monitoring attention.

**Figure 5:**
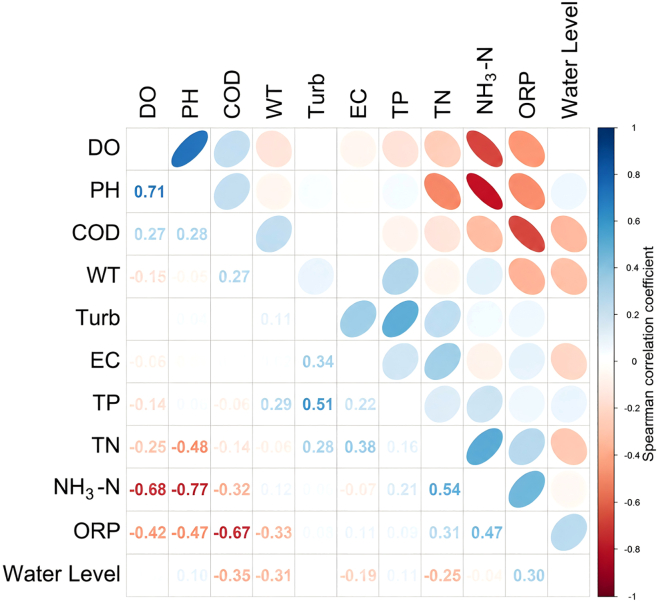
Spearman correlation coefficients among groundwater indicators at Well-2 The color bar represents the Spearman correlation coefficient.

Figure 5 presents a correlation matrix, which visually represents the correlation coefficients between pairs of indicators at Well-2. The color intensity indicates the strength of the correlation. Darker colors represent stronger correlations. There is a strong negative correlation (−0.67) between NH_3_-N and ORP. Some indicators, including COD, water temperature, turbidity, EC, and water level showed no significant correlation, as indicated by the light colors and circular shapes.

By analyzing the indicator correlations across all eight wells, it can be found that there are indicators with high or low correlation degrees at different sites, and the monitoring indicators can be optimized to NH_3_-N, turbidity, COD, water level, and pH by removing the most strongly correlated indicators, which are generally consistent with the conclusion obtained from the NIQR analyses.

Optimization of the monitoring indicators revealed that NH_3_-N, turbidity, COD, water level, and pH were the most effective online monitoring indicators in the park, highlighting their importance for online monitoring. The optimization result provides useful guidance for further improvement of the park’s groundwater environment monitoring system.

It should also be emphasized that the optimized indicator set represents an online-monitoring indicator set rather than a complete characteristic-pollutant inventory for the chemical industrial park. The selected indicators were restricted to parameters that can be measured continuously and reliably at hourly resolution. Heavy metals and trace organic pollutants may be important site-specific contaminants, but they usually require targeted laboratory analysis and may not show stable correlations with conventional online indicators. Therefore, the optimized online indicators should be used to detect hydrodynamic disturbance, general water-quality changes, and possible abnormal events, while periodic monitoring of heavy metals, volatile/semi-volatile organic compounds, and other characteristic pollutants remains necessary for comprehensive groundwater risk assessment.

### Frequency optimization

#### Downsample results

By employing time-domain downsampling, three metrics were used to evaluate the extent of information loss, including MAE, RMSE, and R^2^. To avoid artificial smoothing and potential underestimation of reconstruction error, downsampled series were reconstructed using linear interpolation within the valid temporal range, and no forward or backward filling was applied to boundary values during MAE, RMSE, and R^2^ calculation. This evaluation framework was used to identify an acceptable monitoring interval that balances information fidelity and monitoring efficiency. The analysis was conducted for all monitoring wells and all monitored indicators in the park.

**Figure 6:**
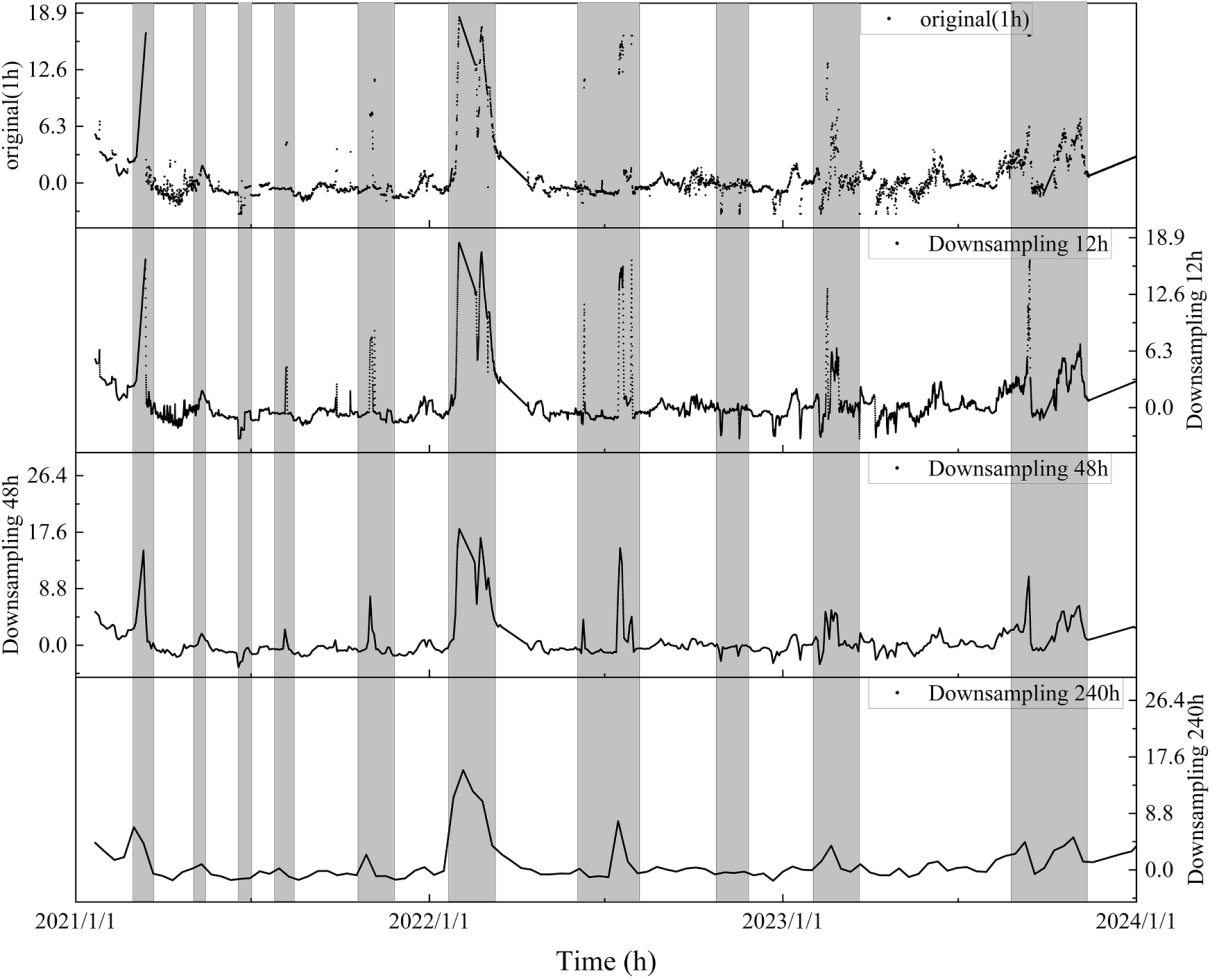
Different downsampling times of COD in Well-5 Temporal information is progressively lost as the downsampling interval increases. Values on the vertical axis are shown as robust-scaled values for visualization.

Figure 6 shows a representative comparison of the COD time series at Well-5 for the original hourly data and the original hourly series, and the series downsampled at 12-, 48-, and 240-h intervals. The significant short-term variations in COD gradually disappeared as the downsampling interval increased.

**Figure 7:**
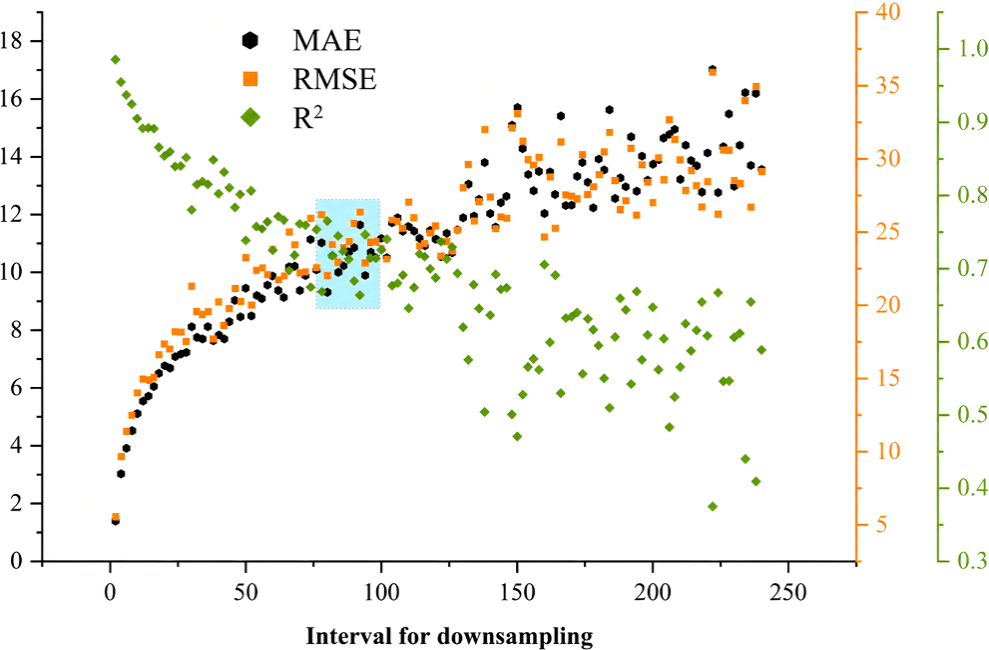
Error metrics for downsampling COD data series at Well-5: MAE, RMSE, and R^2^ across intervals of 2–240 h

Figure 7 shows the results of MAE, RMSE, and R^2^ between the original COD data series and the downsampled COD series from 2 to 240 h at 2 h intervals at Well-5. The interval of 50–100 h was identified as the optimal range for all three metrics. Within this range, both RMSE and MAE are low, while R^2^ is at its highest, indicating strong reconstruction performance with minimal error. As the downsampling interval increased, reconstruction fidelity changed nonlinearly. The 50 to 100 h range provided a balance between information preservation and monitoring reduction, with relatively low MAE/RMSE and acceptable R^2^. Beyond this range, reconstruction performance deteriorated because short-term variations were increasingly lost. This suggests that increasing the interval initially reduces errors, but exceeding 100 h may substantially reduce reconstruction fidelity due to data loss.

The optimized online monitoring frequencies were determined through downsampling analysis for 10 monitored indicators across all monitoring wells in the park, excluding TN (Table 2). Because no unified international standard currently exists for groundwater monitoring frequency, the recommended intervals should be interpreted in relation to site-specific hydrogeological conditions, groundwater dynamic characteristics, and monitoring objectives.

**Table 2:** Optimized monitoring intervals for each well-parameter combination

	NH_3_-N	TP	WL	WT	EC	ORP	pH	Turb	DO	COD
Well-1	369	497	475	717	717	83	211	161	140	30
Well-2	541	29	29	720	719	423	504	91	259	106
Well-3	29	29	29	720	442	82	29	720	296	68
Well-4	29	29	123	719	719	107	29	60	136	33
Well-5	119	29	117	719	720	63	29	159	207	88
Well-6	720	222	483	720	720	720	81	285	720	426
Well-7	156	130	719	720	191	271	70	80	81	150
Well-8	29	29	719	720	445	31	29	559	720	148

Unit: h

The downsampling-based optimization revealed clear heterogeneity in the optimal monitoring intervals across wells and water-quality variables, indicating that a uniform sampling strategy is not suitable for groundwater online monitoring in the industrial park. The optimization was performed independently for each well-parameter combination, and the results showed that the optimal interval was strongly site- and parameter-specific. In general, WT exhibited highly consistent and long optimal intervals across all wells (717–720 h), suggesting stable temporal behavior and limited dependence on high-frequency observations. EC also showed relatively long optimal intervals in most wells (442–720 h), indicating that its dominant temporal dynamics could still be preserved under reduced monitoring frequency.

In contrast, NH_3_-N, TP, pH, and ORP generally required shorter monitoring intervals in several wells, implying stronger short-term variability and a higher sensitivity to frequency reduction. In particular, TP and pH reached the minimum optimized interval of 29 h in five and four wells, respectively, suggesting that coarse sampling could obscure rapid fluctuations or short-duration pollution signals. Strong spatial variability was also evident. For example, Well-6 showed long optimal intervals for multiple variables, indicating relatively stable water-quality dynamics, whereas Well-2 to Well-5 frequently exhibited shorter intervals (29–123 h) for several parameters, implying a greater need for high-frequency monitoring. Variables such as WL, Turb, and DO displayed especially pronounced inter-well differences, further confirming that the dominant temporal scale of groundwater quality is not uniform across sites.

These results suggest that the optimized interval reflects the temporal complexity of each variable rather than simply its concentration level. A shorter optimal interval therefore indicates a stronger dependence on high-frequency data for preserving event-related information. This is consistent with Behmel et al. (2016), who emphasized that water-quality monitoring strategies should jointly consider monitoring objectives, variables, locations, and temporal resolution rather than applying a one-size-fits-all design.^17^ Therefore, the proposed framework provides a practical data-driven basis for adaptive monitoring, in which relatively stable variables such as WT and EC can be monitored at lower frequency, while more dynamic indicators such as NH_3_-N, TP, pH, and ORP should be prioritized for higher-frequency surveillance and early warning.

#### Predictive validation of the optimized sampling frequency

To assess whether the optimized monitoring intervals retained predictive skill, Ridge Regression and Random Forest Regression were applied as independent predictive-validation tools to datasets generated at different sampling frequencies. These models were not intended to serve as a complete early-warning algorithm in this study; instead, they were used to test whether reduced-frequency monitoring retained sufficient temporal information for downstream prediction and potential future data-driven warning applications. For each target variable, the data were split chronologically into training, validation, and test sets in a 6:2:2 ratio. The chronological split was retained to avoid information leakage caused by random temporal mixing. Predictive performance at the optimized coarse sampling interval (Kopt) was compared with that of the 1 h baseline using R^2^, RMSE, and MAE. Specifically, R^2^ loss was defined as ΔR^2^ = R_Kopt_^2^−R_1h_^2^, and RMSE and MAE retention were calculated as RMSE_1h_/RMSE_Kopt_ and MAE_1h_/MAE_Kopt_, respectively. To facilitate comparison across temporal scales, Kopt values were grouped into 24–72 h, 72–168 h, and >168 h.

**Figure 8:**
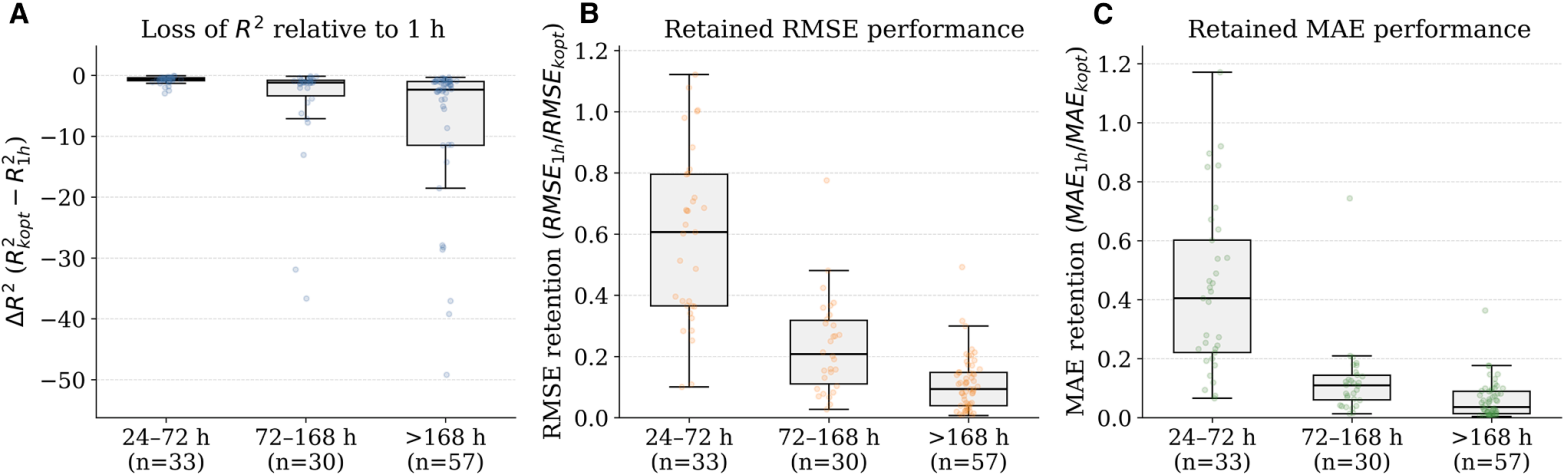
Changes in predictive performance under increasingly coarse optimized sampling intervals

Figure 8 shows the distributions of ΔR^2^, RMSE retention, and MAE retention across the three Kopt groups. Predictive skill declined progressively with increasing Kopt. The distribution of ΔR^2^ shifted toward more negative values from the 24–72 h group to the >168 h group, indicating increasing loss of explained variance relative to the 1 h baseline. Consistent with this pattern, both RMSE and MAE retention decreased as Kopt increased, showing reduced preservation of predictive accuracy under coarser sampling.

Among the three groups, the 24–72 h interval retained the highest predictive skill, whereas substantial degradation was observed for 72–168 h and >168 h. Using an error tolerance threshold of 1.5 times the 1 h baseline, 45.5% of cases in the 24–72 h group remained within the acceptable range, compared with only 3.3% in the 72–168 h group and 0% in the >168 h group.

Together, these results show that predictive skill decreased systematically as the optimized sampling interval increased. Moderate coarse sampling (24–72 h) could still retain useful predictive information in some cases, whereas predictive performance deteriorated sharply beyond 72 h and was largely lost when Kopt exceeded 168 h. These results support the use of predictive modeling as a validation layer for monitoring-frequency optimization. However, the development of fully automated abnormal-event classification and adaptive machine-learning warning thresholds would require independently labeled event records, operational alarm verification, and prospective validation. Therefore, such intelligent warning models are treated as a future extension rather than the central objective of the present study.

To examine whether the chronological 6:2:2 split was biased by seasonal coverage, an additional seasonal sensitivity analysis was conducted by dividing the test period into wet-season (April–September) and dry-season (October–March) subsets. Both Ridge Regression and Random Forest showed consistent performance-degradation patterns across the two seasonal subsets as Kopt increased (Table S3). In both wet and dry seasons, moderate optimized intervals generally retained more predictive information than coarser intervals, whereas predictive performance deteriorated more strongly when Kopt increased beyond longer temporal scales. These results indicate that the predictive-validation results were not dominated by one specific hydrological season. Because the >168 h group contained fewer valid seasonal cases, this group was interpreted cautiously.

### Early warning threshold

Because groundwater quality in chemical industrial parks is often affected by long-term background disturbance, the direct use of fixed regulatory standards may produce frequent alarms and reduce the operational value of early warning. Therefore, this study established dynamic early-warning thresholds based on local baseline conditions derived from long-term online monitoring records. For each indicator, the multi-year mean for the same calendar month after excluding abnormal values was used to represent the local baseline trend, whereas the standard deviation was used to represent background fluctuation. The dynamic threshold was calculated as the multi-year same-calendar-month mean ± k times the corresponding same-calendar-month standard deviation, as described in Equation 12 in STAR Methods.

In this study, k was treated as a site- and parameter-specific engineering calibration coefficient rather than a universal theoretical constant. The coefficient was determined by comparing threshold envelopes generated using different k values with historical time series, documented abnormal periods, sensor uncertainty, and the operational requirement of avoiding excessive alarms under chronically disturbed groundwater conditions. Smaller k values increased sensitivity to moderate deviations but could generate frequent alarms during background fluctuations, whereas larger k values reduced excessive alarms but increased the risk of missing moderate abnormal events. Therefore, different k values were adopted for different well-parameter combinations to account for differences in baseline variability, local hydrogeological conditions, disturbance intensity, and sensor noise. Thus, the calibrated k values should be interpreted as operational parameters constrained by site-specific hydrogeological background and monitoring uncertainty, rather than arbitrary fitting factors. To improve reproducibility, the complete calibrated k values used for the dynamic warning thresholds are provided in Table S2.

**Figure 9:**
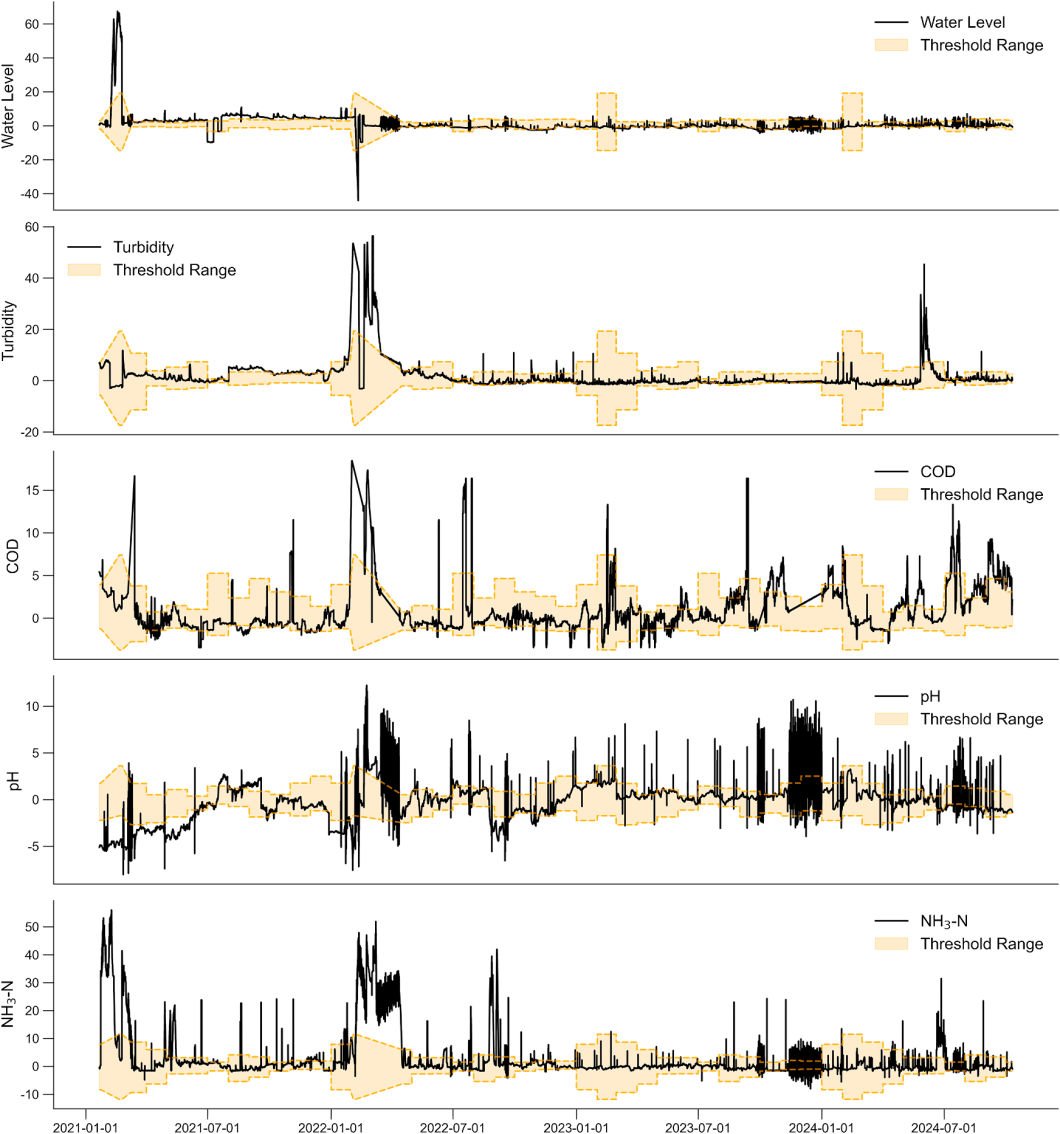
Dynamic early-warning thresholds for representative indicators at Well-5 The shaded light-orange areas indicate the normal fluctuation ranges. Values on the vertical axis are shown as robust-scaled values.

Figure 9 shows representative dynamic early-warning thresholds for multiple indicators at Well-5, including water level, turbidity, COD, pH, and NH_3_-N. The calibrated threshold envelopes captured major abnormal fluctuations while avoiding excessive alarms caused by routine background variability. These results indicate that site-specific calibration of k can improve the operational applicability of dynamic warning thresholds in chronically disturbed groundwater systems.

## Discussion

Continuous groundwater monitoring at hourly resolution provided a substantially more detailed representation of groundwater dynamics than conventional periodic sampling. The approximately 34-month record captured both gradual variations and abrupt anomalies lasting approximately 100–3,360 h, demonstrating the value of long-term high-frequency observations for distinguishing routine background variability from potential anthropogenic disturbances. The coordinated responses of hydrodynamic and hydrochemical variables further indicate that groundwater early warning should be based on multiple complementary indicators rather than on isolated concentration changes.

The indicator-screening results identified COD, NH_3_-N, turbidity, pH, and groundwater level as the most informative online monitoring variables at the study site. However, the marked differences in optimized intervals among wells and parameters demonstrate that a uniform monitoring frequency is unlikely to provide an appropriate balance between information preservation and operational efficiency. Stable variables can potentially be monitored at lower frequencies, whereas indicators with rapid fluctuations require shorter intervals to preserve event-related information. The predictive-validation results further showed that information loss increased as the sampling interval became coarser, particularly beyond 72 h and when the optimized interval exceeded 168 h. Therefore, the reconstruction-based optimized interval should be treated as an upper operational candidate and should be further constrained by the monitoring objective, characteristic event duration, and predictive-information requirements of each well-parameter combination.

Dynamic thresholds based on multi-year same-calendar-month baselines provided a more operationally applicable alternative to the direct use of fixed regulatory standards under chronically disturbed groundwater conditions. Site- and parameter-specific calibration of the coefficient k reduced alarms caused by routine background fluctuations while retaining sensitivity to pronounced deviations. Nevertheless, threshold performance depends on local hydrogeological conditions, contamination history, sensor uncertainty, and the availability of verified abnormal-event records. Consequently, the calibrated coefficients obtained in this study should not be transferred directly to other sites without local validation and recalibration.

Overall, the proposed framework integrates online indicator screening, well- and parameter-specific monitoring-frequency optimization, predictive validation, and dynamic threshold calibration into a practical workflow for adaptive groundwater monitoring. The framework can support more efficient sensor operation and more targeted early-warning management in chemical industrial parks. However, periodic laboratory analysis remains necessary for heavy metals, trace organic contaminants, and other site-specific pollutants that cannot be reliably measured through continuous online monitoring.

### Limitations of the study

This study has several limitations. First, the analysis was based on one chemical industrial park, and the optimized indicator set, monitoring intervals, and calibrated warning thresholds may vary under different hydrogeological settings, industrial structures, and contamination histories. Second, the online indicator system was limited to parameters that can be continuously and reliably measured at hourly resolution. Trace organic pollutants, heavy metals, and other site-specific contaminants were not fully represented by the online sensors and should be assessed through targeted laboratory analysis. Third, the dynamic warning thresholds were calibrated using historical monitoring records and engineering judgment, which improves operational applicability but also introduces site dependence. Finally, the predictive models were used only as validation tools for frequency optimization. Fully automated abnormal-event classification and adaptive threshold updating require independently labeled pollution events, operational alarm records, and prospective validation.

## Resource availability

### Lead contact

Further information and requests for resources should be directed to and will be fulfilled by the lead contact, Zhiren Tian (Email: tianzr@cnemc.cn).

### Materials availability

This study did not generate new physical materials.

### Data and code availability

• Data reported in this paper will be shared by the lead contact upon request.
• The Python scripts used for data preprocessing, indicator screening, monitoring-frequency optimization, predictive validation, and threshold calculation are available from the lead contact upon reasonable request.
• Any additional information required to reanalyze the data reported in this paper is available from the lead contact upon request.

## Acknowledgments

This work was supported by the State Key Laboratory of Soil Pollution Control and Safety, the start-up funds for scientific research of high-level talents in Shenzhen (grant no. 01296126), the 10.13039/501100001809National Natural Science Foundation of China (grant no. 41931292), and MEE Key Laboratory of Integrated Surface Water-Groundwater Pollution Control. The authors are grateful to the editor and the reviewers for their constructive comments and suggestions that significantly improved this manuscript.

## Author contributions

S.Y.: conceptualization, funding acquisition, investigation, methodology, writing – original draft. P.H.: data curation, formal analysis, investigation, validation. Y.D.: data curation, formal analysis. Y.L.: formal analysis, visualization. H.H.: resources, supervision, funding acquisition. Z.T.: project administration, supervision, funding acquisition, writing – review and editing.

## Declaration of interests

The authors declare no competing interests.

## STAR★Methods

### Key resources table

**Table:** 

REAGENT or RESOURCE	SOURCE	IDENTIFIER
**Others**		

VLWS-505 Black-Odor Water Quality Automatic Monitoring Station	Zhejiang Veelang Environmental Technology Co., Ltd.	VLWS-505
TNP-4200 Online Total Phosphorus/Nitrogen Analyzer	Shimadzu Corporation	TNP-4200
TOC-4200 Online Total Organic Carbon Analyzer	Shimadzu Corporation	TOC-4200

**Software and algorithms**		

Python	Python Software Foundation	Version 3.10.15
pandas	Open-source package	Version 2.3.3
numpy	Open-source package	Version 2.2.6
scipy	Open-source package	Version 1.15.3
scikit-learn	Open-source package	Version 1.7.2
matplotlib	Open-source package	Version 3.9.1
kneed	Open-source package	Version 0.8.5

### Method details

NIQR was selected because it provides a robust and unit-independent measure of temporal variability that is less sensitive to extreme values than variance-based statistics, making it suitable for heterogeneous groundwater indicators. Spearman correlation was adopted to identify redundancy because the monitored variables were not guaranteed to follow normal distributions and may contain nonlinear monotonic relationships. Downsampling with reconstruction-based error evaluation was used because the objective of this study was not merely to reduce data volume, but to quantify how much temporal information would be lost under coarser monitoring intervals. Ridge regression and random forest regression were then introduced as complementary validation models, representing linear and nonlinear predictive structures, respectively, to test whether the optimized frequency retained information useful for downstream prediction.

All data preprocessing, statistical analyses, and predictive modeling were conducted in Python (version 3.10.15) using the packages pandas, numpy, scipy, scikit-learn, and matplotlib.

#### Data preprocessing

Data gaps were identified using timestamp discontinuities, power-outage records, instrument-maintenance logs, and system-quality flags. Short gaps caused by temporary power failure or data-transmission interruption were filled using linear interpolation when the gap length did not exceed 6 consecutive hours. Longer gaps were not interpolated and were excluded from analyses requiring continuous time-series reconstruction. Boundary values generated during downsampling reconstruction were treated separately from raw-data gaps. During downsampling reconstruction, linear interpolation was applied only within the temporal range constrained by available downsampled observations. No forward or backward filling was used for boundary values; metric calculation was performed only on time points where both the original and reconstructed series had valid values.

Outliers and invalid records were removed according to both operational records and statistical screening. Records were excluded when they: (i) were collected during sensor calibration, maintenance, or power failure; (ii) exceeded the manufacturer-specified measurement range; (iii) represented physically impossible values, such as negative concentrations; (iv) appeared as isolated spikes inconsistent with adjacent observations and unsupported by simultaneous changes in related indicators; or (v) were flagged as invalid by the online monitoring system.

Measurement uncertainty was evaluated using manufacturer specifications, routine calibration records, and periodic laboratory comparison tests. The main uncertainty sources included sensor drift, probe fouling, matrix interference, environmental disturbance during *in situ* monitoring, and temporary instrument malfunction. The quantitative measurement ranges, accuracy/resolution, calibration practices, and major uncertainty sources of the online sensors are summarized in Table S1.

#### Robust scaling for visualization

To protect the confidentiality of raw concentration values while preserving temporal patterns, all monitored indicators shown in the figures were transformed using robust scaling. For each indicator, the robust-scaled value was calculated as:

(Equation 1) $$\begin{array}{c} x_{\text{scaled}}=\frac{x-\;\text{median}\left(x\right)}{\text{MAD}\left(x\right)} \end{array}$$

where x is the original observation, median(x) is the median of the corresponding indicator series, and MAD is the median absolute deviation. This transformation reduces the influence of extreme values and allows indicators with different units and concentration ranges to be compared on a common relative scale. Robust scaling was used only for visualization and trend interpretation; statistical analyses and monitoring-frequency optimization were performed according to the procedures described in the corresponding STAR Methods subsections.

#### Normalized interquartile range and correlation analysis

In this study, the normalized interquartile range (NIQR) was used to identify the most informative indicators for online groundwater monitoring, and Spearman’s rank correlation coefficient was used to reduce redundancy among indicators by evaluating the similarity of their temporal variation patterns. Together, these two methods enabled the selection of indicators with both high variability and low interdependence.^34^ The NIQR was used as a robust measure of temporal variability for each indicator. It was calculated as:

(Equation 2) $$\begin{array}{c} {\text{NIQR}}_{j}=\frac{Q_{3,j}-Q_{1,j}}{{\bar{x}}_{j}} \end{array}$$

where Q_1,j_ and Q_3,j_ are the first and third quartiles of indicator j, respectively, and ${\bar{x}}_{j}$ is the median value of that indicator. By normalizing the interquartile range with the median, the variability of indicators with different units and value ranges could be compared on a common basis. Compared with variance-based statistics, NIQR is less sensitive to extreme values and is therefore suitable for groundwater monitoring data that may contain occasional abnormal fluctuations.

To further optimize the indicator set, redundancy among indicators was evaluated using Spearman’s rank correlation coefficient. This non-parametric method was selected because it does not require the assumption of normality and is suitable for identifying monotonic associations in environmental time-series data. The coefficient was calculated as:

(Equation 3) $$\begin{array}{c} \rho_{s}=1-\frac{6\sum_{i=1}^{n}d_{i}^{2}}{n\left(n^{2}-1\right)} \end{array}$$

where ρ_s_ is Spearman’s rank correlation coefficient, d_i_ is the difference between the ranks of the i-th paired observation for two indicators, and n is the number of paired observations. In the presence of tied values, average ranks were assigned before calculation. Indicators showing strong redundancy based on Spearman correlation were considered to contain overlapping information and were therefore further screened in combination with NIQR results.

#### Downsampling-based optimization

Time-domain downsampling was used to identify the optimal monitoring frequency for groundwater online monitoring in the industrial park. Errors for data series at different sampling frequencies were computed by comparison with the original long-term hourly data.^35^ A downsampling-based optimization framework was developed to determine the optimal monitoring interval for groundwater quality variables across multiple monitoring stations. The objective of this method is to reduce monitoring frequency while preserving the essential temporal dynamics and pollution-event information contained in the original high-resolution dataset.^36^ Let the original hourly monitoring time series be denoted as y(t), where t = 1,2, …,n. For a given sampling interval K (in hours), a downsampled sequence y_K_(n) is constructed by retaining observations at every K-th time step. The downsampling operation can be expressed as:

(Equation 4) $$y_{K}\left(n\right)=f\left(y\left(t\right),K,\delta \right)$$

where f denotes the downsampling operator, and δ∈[0,1, …,K−1] represents the sampling offset. To reduce the influence of phase dependency associated with different starting points, multiple offsets were considered for each sampling interval K. Specifically, when K was small, all possible offsets were evaluated; when K was large, a fixed number of offsets were uniformly sampled across the interval. For each offset-specific downsampled series, the retained observations were mapped back to the original time axis, and the hourly-resolution series was reconstructed using linear interpolation within the temporal range supported by the downsampled observations. No forward or backward filling was applied to boundary values, because such filling could introduce artificial smoothing at the edges of the reconstructed series. The reconstructed series was then compared with the original hourly observations only at time points where both series contained valid values.

The discrepancy between the original series y(t) and the reconstructed series ${\hat{y}}_{K}$ was quantified using three statistical metrics: mean absolute error (MAE), root-mean-square error (RMSE), and the coefficient of determination (R^2^). These metrics are defined as:

(Equation 5) $$\begin{array}{c} MAE\left(K\right)=\frac{1}{n}\sum_{i=1}^{n}|y_{i}-{\hat{y}}_{K,i}| \end{array}$$

(Equation 6) $$\begin{array}{c} RMSE\left(K\right)=\sqrt{\frac{1}{n}\sum_{i=1}^{n}{\left(y_{i}{\hat{y}}_{K,i}\right)}^{2}} \end{array}$$

(Equation 7) $$\begin{array}{c} R^{2}\left(K\right)=1-\frac{\sum_{i=1}^{n}{\left(y_{i}{\hat{y}}_{K,i}\right)}^{2}}{\sum_{i=1}^{n}{\left(y_{i}\bar{y}\right)}^{2}} \end{array}$$

where y_i_ is the observed value at time step i, ${\hat{y}}_{K,i}$ is the reconstructed value at the same time step, and $\bar{y}$ is the mean of the original series.

Because these metrics differ in magnitude and optimization direction, a normalization procedure was applied prior to aggregation. R^2^ is positively oriented, whereas MAE and RMSE are negatively oriented. Therefore, MAE and RMSE were transformed into positively oriented metrics through normalization and inversion. A composite performance score for each sampling interval K was then defined as:

(Equation 8) $$\begin{array}{r} \text{Score}\left(K\right)=\frac{R_{\text{norm}}^{2}\left(K\right)+\left(1-\text{MA}E_{\text{norm}}\left(K\right)\right)+\left(1-\text{RMS}E_{\text{norm}}\left(K\right)\right)}{3} \end{array}$$

where $R_{\text{norm}}^{2}\left(K\right)$, MAE_norm_(K) and RMSE_norm_(K) denote the min–max normalized values of the respective metrics.

As the sampling interval K increases, the composite score generally decreases due to progressive information loss. To identify the optimal monitoring interval, the knee point of the Score(K) curve was detected using the KneeLocator algorithm.^37^ The corresponding value of K at the knee point was selected as the optimal monitoring interval, representing the largest sampling interval before a rapid deterioration in reconstruction performance occurs. If no clear knee point was identified, the interval corresponding to the maximum composite score was selected as a fallback.

To improve computational efficiency while maintaining resolution at short time scales, a non-uniform candidate interval set was adopted. Specifically, shorter intervals were evaluated at fine resolution, whereas longer intervals were sampled more sparsely. In addition, an early stopping strategy was implemented: if the average R^2^ remained below a predefined threshold for several consecutive larger intervals, further evaluation was terminated, as reconstruction performance was unlikely to improve at coarser scales.^38^ This framework was applied independently to each monitoring station and each water quality parameter.^17^ By comparing fluctuations in the curve post-frequency optimization, the optimal time intervals for monitoring can be identified, allowing for reduced monitoring frequency without sacrificing accuracy.^36^^,^^39^

#### Ridge regression and random forest regression

To further validate the optimized monitoring frequency, Ridge Regression (RR) and Random Forest Regression (RFR) were employed as independent predictive-validation tools. The purpose of this analysis was to assess whether the optimized sampling frequencies retained sufficient temporal information for downstream prediction, rather than to construct a complete machine-learning-based early-warning model.^40^^,^^41^^,^^42^

For each monitoring well and target parameter, lagged features were constructed from historical observations, and the target value at the next time step was used as the prediction output. The datasets were divided chronologically into training, validation, and test sets to avoid information leakage.^43^ To evaluate whether the chronological split was affected by seasonal coverage, an additional seasonal sensitivity analysis was performed. The test period was divided into wet- and dry-season subsets according to local hydrological conditions, and predictive-performance changes from the 1 h baseline to the optimized sampling interval (Kopt) were recalculated separately for each subset. This analysis was used to examine whether the assessment of model generalization was dominated by one specific hydrological season. The detailed seasonal sensitivity results are provided in Table S3.

For RR, the prediction model can be expressed as

(Equation 9) $$\begin{array}{c} \hat{y}=\beta_{0}+\sum_{j=1}^{p}\beta_{j}x_{j} \end{array}$$

where x_j_ denotes the input features, β_j_ denotes the regression coefficients, and p is the number of predictors. To reduce overfitting caused by multicollinearity, RR introduces an L2 regularization term, and the objective function is defined as

(Equation 10) $$\begin{array}{c} \min_{\beta }\left\{\sum_{i=1}^{n}{\left(y_{i}{\hat{y}}_{i}\right)}^{2}+\lambda \sum_{j=1}^{p}\beta_{j}^{2}\right\} \end{array}$$

where n is the number of samples and λ is the regularization parameter.

For RFR, the final prediction is obtained by averaging the outputs of multiple regression trees:

(Equation 11) $$\begin{array}{c} \hat{y}=\frac{1}{B}\sum_{b=1}^{B}T_{b}\left(x\right) \end{array}$$

where B is the number of trees and Tb(x) is the prediction of the b-th regression tree for input x. By combining bootstrap sampling and random feature selection, RFR can effectively improve model robustness and represent nonlinear interactions among variables.^42^^,^^44^

Model performance was evaluated using R^2^, RMSE, and MAE. If the optimized monitoring frequency retained acceptable predictive performance relative to the original 1 h observations, it was considered effective in reducing monitoring effort while preserving the information required for groundwater quality prediction.^44^ This validation step provides evidence for the information-preservation capacity of the optimized monitoring frequency. The construction of a fully automated machine-learning anomaly-detection model would require independently labeled abnormal events, operational alarm records, and prospective validation, which were beyond the scope of the present study.

#### Warning setting

The setting of groundwater early-warning thresholds in industrial parks remains an issue that requires further study in China. Given the poor quality of groundwater in these areas, the direct application of conventional water-quality standards may lead to overly frequent warnings to overly frequent warnings, thereby losing the significance of early warning. Therefore, thresholds based on local groundwater baseline conditions may capture pollution incidents more accurately.^45^

Based on the analysis of nearly three years of online monitoring data, the multi-year mean for the same calendar month after excluding abnormal values was used to represent the baseline trend term, whereas the corresponding same-calendar-month standard deviation was used to represent the fluctuation term.^46^^,^^47^ The combination of the trend and fluctuation terms was used to define the upper and lower threshold bounds. For each indicator:

(Equation 12) $$\begin{array}{c} T_{m}=\mu_{m}\pm k\sigma_{m} \end{array}$$

where *T*_*m*_ represents the upper and lower dynamic threshold bounds for calendar month m, *μ*_*m*_ is the multi-year mean for the same calendar month after excluding abnormal values, *σ*_*m*_ is the corresponding same-calendar-month standard deviation, and k is a site- and parameter-specific engineering calibration coefficient determined from the historical monitoring data.^47^

In this study, k was treated as a site- and parameter-specific engineering calibration coefficient rather than a universal theoretical constant. The selection of k was based on historical monitoring records, background variability, known abnormal periods, sensor uncertainty, and the operational requirement of avoiding excessive alarms under chronically disturbed groundwater conditions. For each monitoring well and indicator, the threshold envelope was adjusted to cover normal seasonal and background fluctuations while retaining sensitivity to pronounced abnormal changes associated with potential pollution disturbances. Therefore, different k values were adopted for different wells and indicators because baseline variability, local hydrogeological conditions, disturbance intensity, and sensor noise differed among well-parameter combinations. The calibrated k values used in this study are listed in Table S2.

### Quantification and statistical analysis

Indicator screening was based on the normalized interquartile range (NIQR) and Spearman’s rank correlation coefficient. No inferential hypothesis tests were performed in this study. Reconstruction performance during monitoring-frequency optimization was evaluated using mean absolute error (MAE), root-mean-square error (RMSE), and coefficient of determination (R^2^), and these metrics were aggregated into a normalized composite score for knee-point detection. Predictive validation using ridge regression and random forest regression was assessed using R^2^, RMSE, and MAE. Dynamic warning thresholds were calculated using same-calendar-month baseline statistics and site-specific engineering calibration of the standard-deviation multiplier k. Sensor uncertainty was summarized based on manufacturer specifications, routine calibration records, and periodic laboratory verification.

For the hourly monitoring dataset, n represents the number of valid hourly observations for each well–parameter combination after data-quality screening. The raw dataset contained 1,794,813 recorded hourly observations before preprocessing. Following data-quality screening and linear interpolation of short gaps, the numbers of analysis-ready observations for each indicator are reported in Table 1. For predictive validation, n represents the number of valid cases included in each optimized sampling-interval group, as reported in the corresponding figure legends and supplemental tables. Center and dispersion were described using the median, interquartile range, mean, and standard deviation where appropriate. All data preprocessing, statistical analyses, and predictive modeling were conducted in Python 3.10.15 using pandas, numpy, scipy, scikit-learn, matplotlib, and kneed.

### Additional resources

No additional resources are associated with this study.

## Supplemental information

Document S1. Tables S2 and S3

Table S1. Sensor-performance specifications, calibration and quality-control procedures, and major uncertainty sources for the online monitoring instruments used in this study
