# Supplementary material for: Optimizing online groundwater monitoring in industrial parks using long-term high-frequency data
Source: iScience. 2026 Aug 6;29(8):117042. doi: 10.1016/j.isci.2026.117042 (PMC13475684; doi:10.1016/j.isci.2026.117042)
Supplement: Supplementary file 1 [file mmc1.pdf]

iScience, Volume *29*

Supplemental information

Optimizing online groundwater monitoring in industrial parks using long-term high-frequency data

Shuping Yi, Pizhu Huang, Yi Deng, Yi Liu, Hefei Huang, and Zhiren Tian

Supplemental Tables

Table S2. Calibrated k values used for dynamic warning thresholds across well–parameter combinations.

	Well-1	Well-2	Well-3	Well-4	Well-5	Well-6	Well-7	Well-8
NH3-N	2.2	1.6	2.5	2.3	2.4	1.5	2.4	2.5
TP	2	1.9	2.2	2.5	2	1.7	2.3	2.5
TN		1.6				1.5		
WL	1.7	2	2	1.8	1.5	1.5	1.9	1.6
WT	1.8	1.8	1.7	2	2.3	1.9	2.2	2.1
EC	1.6	1.5	1.5	1.5	1.5	1.5	2	1.5
ORP	1.5	1.6	1.6	1.6	1.6	1.6	1.5	1.5
pH	1.7	1.5	2	2.5	2.5	1.8	2.1	2.4
Turb	1.5	1.5	1.5	1.9	2.3	1.7	1.8	1.5
DO	1.5	1.5	1.5	1.9	1.5	1.5	1.7	1.5
COD	1.5	1.6	1.6	2.1	1.6	1.6	1.9	1.6

The threshold coefficient k was calibrated for each well–parameter combination based on historical baseline variability, documented abnormal periods, sensor uncertainty, and the operational requirement of avoiding excessive alarms. Smaller k values indicate higher sensitivity to moderate deviations, whereas larger k values were used for indicators or wells with greater background variability or higher noise.

Table S3. Seasonal sensitivity validation of predictive performance under the chronological 6:2:2 split.

Model	K_{opt} group	Season	median ΔR^2	Mean ΔR^2	median RMSE retention	mean RMSE retention	Median MAE retention	Mean MAE retention
Random Forest	24–72 h	Dry	-0.608	-0.680	0.384	0.509	0.192	0.262
		Wet	-0.590	-0.649	0.680	0.664	0.399	0.414
	72–168 h	Dry	-1.194	-1.472	0.172	0.201	0.073	0.091
		Wet	-1.119	-2.184	0.278	0.316	0.123	0.152
	>168 h	Dry	-2.070	-2.577	0.196	0.245	0.095	0.160
		Wet	-1.316	-2.008	0.174	0.191	0.091	0.110
Ridge	24–72 h	Dry	-0.378	-0.623	0.420	0.508	0.290	0.310
		Wet	-0.567	-0.605	0.591	0.627	0.399	0.434
	72–168 h	Dry	-1.053	-1.460	0.188	0.168	0.065	0.072
		Wet	-1.020	-1.309	0.313	0.344	0.121	0.151
	>168 h	Dry	-1.476	-1.444	0.113	0.137	0.073	0.071
		Wet	-1.177	-1.557	0.140	0.163	0.073	0.080

ΔR^2 was calculated as $R^{2}Kopt - R^{2}1h$. RMSE retention and MAE retention were calculated as RMSE1h/RMSEKopt and MAE1h/MAEKopt, respectively. Wet and dry seasons were defined according to local hydrological conditions, with April–September classified as the wet season and October–March as the dry season. The >168 h group included a relatively small number of valid seasonal cases; therefore, the corresponding seasonal comparisons should be interpreted with caution.
